# Stable cognitive performance while adapting to intermittent fasting: A randomised controlled trial

**DOI:** 10.1177/13591053251351204

**Published:** 2025-07-23

**Authors:** Christoph Bamberg, Julia Reichenberger, Jens Blechert

**Affiliations:** 1 Paris Lodron University of Salzburg, Austria; 2 Ludwig-Maximilian University Munich, Germany

**Keywords:** ambulatory assessment, concentration, emotion, intermittent fasting, time restricted eating

## Abstract

Intermittent fasting (IF) is a popular health regimen for weight loss and overall health. Yet, potential negative side effects on cognition and mood could hinder its adoption. Such side effects, however, have so far only been studied in short term exposures to IF. We tested whether fasting impairs cognitive performance and mood when adapting to 16-hour, breakfast-skipping IF over 10 days in a randomised, waitlist-controlled trial (*N* = 122). Cognitive performance was repeatedly assessed online via standardised psychological tasks and subjective concentration and mood measured twice daily via smartphone questionnaires. Bayesian multi-level modelling revealed that fasting participants did not have lower cognitive performance or mood compared to control participants. However, participants felt less concentrated while fasting before noon, compared to after breaking the fast in the afternoon. Thus, 16-hour IF does not cause cognitive performance or mood deficits, removing a potential concern in its use as a health intervention.

## Introduction

Intermittent Fasting (IF) is a widely practiced eating regimen (IFIC, 2020). It is popular for its supposed health benefits (Mattson, 2022; Patterson and Sears, 2017) and is used to treat overweight and obesity with comparable weight-reduction success as classical dieting (Alhamdan et al., 2016; Barnosky et al., 2014; Harvie and Howell, 2017; Johnstone, 2015; Schübel et al., 2018). Yet, it does not come without costs: side effects like higher risk of binge-eating (Stockman et al., 2018) and association with eating-disorder symptoms have been reported (Cuccolo et al., 2022). Also, potential cognitive detriments from IF are important to consider to judge its feasibility as a lifestyle or health intervention, given the importance of cognitive performance in daily life (Lövdén et al., 2020). Moreover, negative effects of IF on practitioners’ mood could impair the adherence to the intervention, challenging its practical significance. Thus, we assessed whether interval fasting by daily breakfast skipping leads to reductions in cognitive performance and worse mood.

Several studies documented effects of single fasting sessions on cognitive variables including reduced subjective work performance and higher distractibility in women doing Alternate Day Fasting (ADF; Appleton and Baker, 2015), lower cooperative behaviour in an economic game in a group of men fasting for 16 hours (Bamberg et al., 2021) or reduced school performance in breakfast-skipping children (Pollitt et al., 1981). However, other studies did not find differences between fasted and satiated participants in related cognitive performance indicators such as perceptual accuracy for food-unrelated words (Radel and Clément-Guillotin, 2012), inhibitory control measured on a Stroop task (Stewart and Samoluk, 1997) or set-shifting abilities (Piech et al., 2009). Effects on mood such as increased irritability but also higher sense of control in women fasting for 18 hours have been reported (Watkins and Serpell, 2016). Furthermore, self-reported energy and wakefulness were higher when consuming breakfast after an overnight fast versus a calorie-free placebo in another study (Maridakis et al., 2009). Yet, several studies did not find differences in self-report measures like depression, anxiety and stress between fasted and satiated participants (Bartholdy et al., 2016; Bolton et al., 2014; Green et al., 1997).

Importantly, most of these studies did not track the outcomes over several days to properly assess how these effects develop over the adaptation period to IF. Effects may especially change over the first few days given that the novelty of fasting can influence self-report outcomes in itself (Ma et al., 2021). Yet, studies measuring cognitive or psychological effects of fasting for several days are rather scarce due to the demanding nature of such protocols. Three studies tested continuous, not intermittent, fasting for several days. For instance, the mood of 13 participants who abstained entirely from eating for 10 days followed a U-shaped trajectory during this period (Yang et al., 2021). Yet, their performance on a psychomotor vigilance task and shift number task showed no significant changes. In contrast, cognitive flexibility and set-shifting performance increased in nine amateur weightlifters after a 48-hour fast, while working memory remained unaffected (Solianik et al., 2016). Participants’ anger but no other mood state was higher while fasting. Self-reported concentration, energy and emotional balance increased from before to during 7 days of uninterrupted fasting for 13 participants (Michalsen et al., 2003). Notwithstanding, these studies lacked control groups and had only small samples, showing the necessity of larger investigations with fasting protocols spanning several days.

Recently, some studies started testing effects of intermittent fasting protocols on cognitive performance and mood. For instance, a group of individuals with high cholesterol did not change in cognitive performance after twice-weekly 24 hours ADF for 4 weeks, compared to baseline (Bartholomew et al., 2021). Daily IF of 16 hours for 2 months did also not lead to pre-mid-post changes in cognitive performance or mood in a group of 76 healthy volunteers (Batorek and Hromatko, 2024). While these studies measured IF and its effects prospectively over time at defined time points, any potential dynamic trajectory of IF-effects during metabolic, cognitive and psychological adaptation to IF remains opaque in these studies.

In sum, there is a gap between studies that assessed effects of single fasting sessions and studies with more intense protocols but small sample sizes or few measurement points. This dissociation is important as it might drive divergence of findings: studies with single fasting sessions overall found more detrimental effects than studies with continuous and longer periods (describing no or even positive effects on cognitive performance). An unpublished meta-analysis of studies testing effects of IF on objective cognitive performance summarised this, finding that the negative effect of fasting decreased with longer fasting durations, indicating a potential adaptation to IF (Bamberg and Moreau, 2025). We intended to fill this gap by studying a 10 day IF intervention with daily outcome measurements with high statistical power in a randomised controlled trial with sample size of 120 participants. We measured cognitive performance through standardised psychological tasks and self-report, to cover different manifestations. We also recorded mood as an outcome measure and potential moderator for effects on cognitive performance. The study was run remotely, in the participants environment, to increase the ecological validity and feasibility of the repeated cognitive testing. We aimed for an isocaloric IF intervention by emphasising potential health but not weight effects and by encouraging afternoon compensatory calory intake. Thus, our findings generalise to different use cases of IF.

Based on the negative findings from single fasting session studies, we expected that fasted participants display reduced cognitive performance and worse mood in the beginning of the intervention. We expected this difference to be reversed at the end of the intervention with fasted participants showing higher cognitive performance and better mood than the control group, based on studies with fasting durations over several days. These hypotheses and methods were preregistered prior to data collection on the Open Science Framework (https://doi.org/10.17605/OSF.IO/968MG).

## Methods

### Participants

Participants were recruited from German and Austrian universities and via social media. Recruitment materials emphasised that intervention goal was not weight-loss but overall health. Eligibility criteria included German language proficiency, age between 18 and 45 years and a BMI between 18.5 and 30 kg/m^2^. Exclusion criteria were self-reported eating disorders within the past 3 years, malnutrition, metabolic diseases (e.g. diabetes mellitus) or an inability to fast for 16 hours. Participants also could not have previously engaged in prolonged fasting. Thus, all participants were fasting-naïve since prior experience may influence their response to the intervention (Ma et al., 2021). Compensation included 50€–60€, depending on protocol adherence or 6 course credits for psychology students at the [anonymised for peer-review]. We aimed for a sample size of *N* = 60 in both groups based on a simulation-based a priori power analysis conducted in R using the *faux* package (DeBruine, 2023). Our goal was to obtain β = 0.8 power to detect a medium effect size of 0.3, accepting a false positive rate of α = 0.05. Participants were randomly allocated to the IF intervention group or wait-list control group. The random number list used to determine group assignment was created using a pseudo random number generator implemented in R (see Supplemental Material). Ethical approval was obtained from the ethics review board of the University of Salzburg on 12.07.2023 with approval number EK-GZ 21/2023_2.

### Procedure

Participants were contacted via email. After providing consent and completing preliminary questionnaires, a video call was scheduled to explain the study aims, answer questions, instal the M-Path app (Mestdagh et al., 2023), test a cognitive task for technical issues and inform participants of their assigned condition. The study start was coordinated with participants to ensure compliance and feasibility. The study began with a 4-day baseline period followed by 10 days of IF. Table 1 shows these phases and on what day each measurement was performed. The cognitive tasks and noon smartphone questionnaires could be done by participants between 11:00 AM and 2:00 PM. This response window was chosen so that participants can integrate the study well into their daily routine. All cognitive tasks and smartphone questionnaires were equal between groups except some additional smartphone questions pertaining to the perceived obstacles and feasibility of the intervention for the fasting group.

**Table 1.** Sampling scheme and intervention.

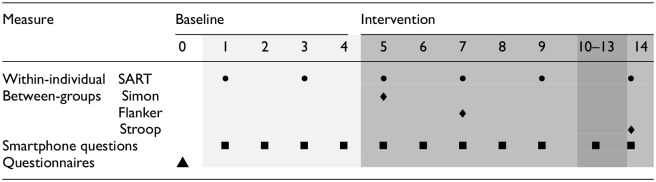

The table shows the days of the intervention and what measurements (signified by the different symbols) were performed for each day. The IF group began fasting on the evening of Day 4, at the end of the baseline period.

#### Intervention

Participants in the IF group fasted for 16 hours daily by skipping breakfast (no food after 8 pm and no food before 12 pm). They were advised to prepare breakfast in advance and consume it later to encourage sufficient calorie intake within the 8-hour eating window, since weight-loss was not the aim of the intervention. Participants were reminded to stay hydrated with non-caloric fluids, to fast only as much as comfortably possible and to honestly report their fasting duration, as one study aim was to assess the feasibility of IF in everyday life. The wait-list control group received information on how to follow the IF protocol at the end of the study and could then begin fasting without supervision.

### Materials

All measurements and instructions were performed and delivered online.

### Cognitive tasks

Within-individual cognitive performance was assessed using the Sustained Attention to Response Task (SART) on 6 days, monitoring changes over time. For a more comprehensive cognitive profile testing between groups, we added three single session Go-No-Go tests. Tasks were programmed in PsychoPy (Peirce et al., 2019; Version 2022.2.5) and delivered via Pavlovia through participants’ web browsers (Bridges et al., 2020). Our rationale for this combination was twofold. First, we aimed to track cognitive performance consistently within individuals by repeating the same task throughout the study. However, we recognised that this approach could lead to training effects, where participants improve over time and approach a performance ceiling, reducing variability and limiting our ability to detect differences between groups (Maridakis et al., 2009; Thomas et al., 2016). To address this potential limitation, we supplemented the design with three additional tasks that were administered only once and varied between groups. This ensured that, while we maintained within-subject consistency, we also introduced novel assessments that allowed for clearer between-group comparisons without the confounding effects of repeated exposure.

#### Within-individual SART

The within-individual SART presented a stream of digits (1–9), with one digit (e.g. 1) randomly designated as the No-Go stimulus at the start of each session and appearing in 11% of trials. The remaining digits (e.g. 2–9) served as Go stimuli. Participants were instructed to press the space bar quickly for Go stimuli and withhold responses for the No-Go stimulus. Each trial lasted 1600 ms: the digit appeared centrally for 250 ms, followed by a 50 ms blank screen and a fixation cross for up to 1300 ms or until participants responded. Font size was randomised to prevent reliance on digit shape. Participants could respond until 1000 ms post-stimulus onset. Instructions emphasised speed to encourage errors. Each session included 180 trials across 4 blocks, with 15-second breaks between blocks. The number of commission errors – erroneously responding to a no-go trial – is the main dependent, within-individual variable of cognitive performance

#### Between-group Simon, Stroop and Flanker task

The between-group tasks followed a similar structure. Each task included 240 trials (50% incongruent), with stimuli presented for 1250 ms, followed by an 800 ms fixation cross and a 250 ms blank screen. Responses were allowed during stimulus presentation. Sessions began with practice trials (8 for Simon and Flanker, 12 for Stroop), with feedback provided.

The stimuli differed between the tasks. In the Simon Task, participants responded to the colour of a square (press ‘A’– left side of keyboard for red, ‘L’ -right side of keyboard for blue) presented on the left or right of the screen. For incongruent trials (e.g. a red square on the left), participants had to inhibit location-based responses and press ‘A’ for colour instead.

In the Stroop Task, participants responded to the meaning of colour words (e.g. ‘red’ = left arrow, ‘blue’ = right arrow, ‘green’ = down arrow), ignoring the font colour. For incongruent trials, such as ‘red’ written in blue, they responded to the word meaning, not the font colour.

The Flanker Task presented central arrows (‘<’ or ‘>’) flanked by lateral distracting arrows. Participants responded to the central arrow (press ‘A’ for ‘<’, ‘L’ for ‘>’) while ignoring the flanking arrows (e.g. ‘> > < > >’ or ‘< < > < < ’). The error rates from each task are the between-groups measurements.

#### Smartphone questionnaires

In addition to these objective cognitive performance measures, participants reported their *subjective* experiences via smartphone-based questionnaires across 14 days. Notifications were sent at three fixed time points daily (signal-contingent sampling): morning (6:00–11:00 AM), noon (11:00 AM–2:30 PM), and evening (9:00 PM–12:00 AM). Half-hourly reminders were sent until responses were recorded. Participants also received prompts for fasting (if applicable) and cognitive tasks. Fasting group participants answered the noon prompt just before breaking their fast to maximise intervention effects. The questionnaires included closed questions, primarily using visual analogue scales (VAS; 0–100, e.g. ‘not at all’ to ‘very much’) or hour-based formats. Questions referenced the time since the last prompt (e.g. ‘How was your concentration since the last prompt at noon?’).

Questionnaires were delivered via the M-Path app. Table 2 shows the main smartphone questions, a complete list can be found in the Supplemental Material.

**Table 2.** Main smartphone questions.

Question	Time of day		
	Morning	Noon	Evening
Sleep quality	x		
Sleep duration	x		
Sleepiness		x	x
Concentration		x	x
Cognitive task ability		x	x
Mood		x	x
Hunger	x	x	x
Desire to eat		x	x
Meal balance and healthiness			x
Physical activity			x
IF feasibility			x (IF only)
Fasting duration			x (IF only)

Mood was assessed with the items active, happy, relaxed, full of energy, worried, irritated, nervous, depressed.

#### Trait questionnaires

The pre-intervention questionnaires contained questions on demographic and socio-economic status. Furthermore, we asked participants for their expectations regarding intervention effects to be able to control for potential placebo-effects. Participants also responded to several standardised questionnaires (see Supplemental Material).

### Statistical analysis

To ensure that online tasks show the expected cognitive effects (independent of the intervention), we tested whether response times after an error trial are longer than before the error trial (‘post error slowing’) for the within-individual task and whether response times on incongruent trials are longer than on congruent trials for the between-groups tasks. We averaged the self-report items *concentration* and *cognitive task ability* into subjective cognitive performance. The single mood items were averaged to a negative (items *worried, irritated, nervous, depressed*) and a positive mood scale (items *active, happy, relaxed, full of energy*). IF compliance was assessed through self-report (daily fasting duration and time of first/last meal).

We used a Bayesian analysis approach because we expected no differences for some hypotheses, which cannot be tested properly with frequentist null hypothesis significance testing (Rouder et al., 2009; Wagenmakers et al., 2008, 2010). We modelled differences between fasting and control groups through a group * measurement repetition interaction for the within-individuals task or group * days/time of day for the smartphone questionnaire data. Measurement repetition was added as a varying effect, grouped by participants. The between-group cognitive performance models lacked an interaction and only had a varying intercept per participant. The average over the four baseline days was added as a covariate to control for baseline levels. As a result, the intercept in the model is not the baseline value but rather the value at the beginning of the intervention. Bayesian priors were weakly informative (see Supplemental Material for details and sensitivity analysis for main models). The likelihood functions for the data were based on the assumed data generating processes and are reported along the results. Our multilevel models handle missing data by pooling information across participants and time points, allowing us to include incomplete cases – similar to intention-to-treat analysis with multiple imputation. Note that missing data were rare and similarly spread across IF and control group (in control group, 4.24% and in IF group, 5.65% of within-individual tasks were missing), making selective drop out unlikely.

Medians (denoted with *B*) and 95% Credibility Interval (95% CI) of coefficients’ posterior distribution are reported. An effect is substantial if its 95% CI does not contain zero for smartphone questionnaire data or does not contain 1 for odd-scaled objective cognitive performance measures. We report odds instead of log odds for all objective cognitive performance models. Furthermore, we use Bayes Factors to test how likely the data is given a specific model. If not indicated otherwise, we compare a model with the group assignment factor with a Null model without group assignment. A Bayes factor represents the likelihood of the data under Model 1 (e.g. with a factor for group assignment) compared to the Null Model (without the group assignment). A BF_10_ of 3 indicates the data is three times more likely under Model 1, while BF_10_ = 1/3 or BF_01_ = 3 suggests the opposite (Stefan et al., 2019). Bayes Factors are commonly interpreted as follows: BF_10_ = 1–3 (not worth mentioning), BF_10_ = 3–20 (positive evidence), BF_10_ = 20–150 (strong evidence), and BF10 > 150 (very strong evidence; Jeffreys, 1983; Kass and Raftery, 1995).

If the effective sample size for the central tendency or tails of the posterior distribution was below 500 or the R-hat diagnostic above 1.02, the number of iterations per MCMC chain was increased from 2000 to 12,000. Thus, for all smartphone questionnaire models, sampling was run with 12,000 iterations since the diagnostic values for the grouped effects were poor otherwise.

## Results

### Participants, compliance and task sensitivity checks

We enrolled 138 participants between November 2023 and April 2024, of which 17 did not complete the assessment and three were excluded for technical reasons (see Supplemental Figure S1 for a flow chart). The analysed sample consists of 121 individuals (IF *N* = 62, control *N* = 59), of which 98 were female (IF female *N* = 48, male *N* = 14; CG female *N* = 50, male *N* = 9). Participants were on average 26.30 years old (*SD* = 6.29), with a BMI of 23.19 kg/m^2 (*SD* = 2.49 kg/m^2^).

The average reported fasting duration in the intervention group was 15.99 hours (*SD* = 2.08), indicating that participants complied with the instructions. Participants broke their fast at 12 AM on average (*SD* = 2.77 hours) and had their last meal at 07:30 PM (*SD* = 1.88 hours). Importantly, fasted and control participants responded to the noon questionnaires and tasks at a similar time (IF *M* = 12:09 AM, *SD* = 1.13 hours; CG *M* = 12:28 AM, *SD* = 1.3 hours), ruling out the time of day as a factor distinguishing the groups. Fasting participants were substantially hungrier while fasted (*BF_10_* = 2.85 × e^11^; note that the subscript 10 in *BF_10_* indicates evidence in favour of M1 over M0) than controls (IF: *M* = 50.13; *SD* = 26.96; controls: *M* = 38.04; *SD* = 24.96). On average, participants neither had positive nor negative expectations regarding their cognitive performance during fasting (*M* = 51.53, *SD* = 22.79).

As for the cognitive tasks’ successful implementation, participants responded substantially slower on trials after a commission error than on trials leading up to the error (*BF_10_* = 8.64 × e^16^; post-commission error RT *M* = 0.39, *SD* = 0.11; pre-commission error RT *M* = 0.37, *SD* = 0.01), confirming that the task was implemented correctly. For the between-groups tasks, response times were slower on incongruent than on congruent trials, as expected (see Supplemental Material).

### Did fasting affect participants’ objective cognitive performance over time?

**Figure 1.**
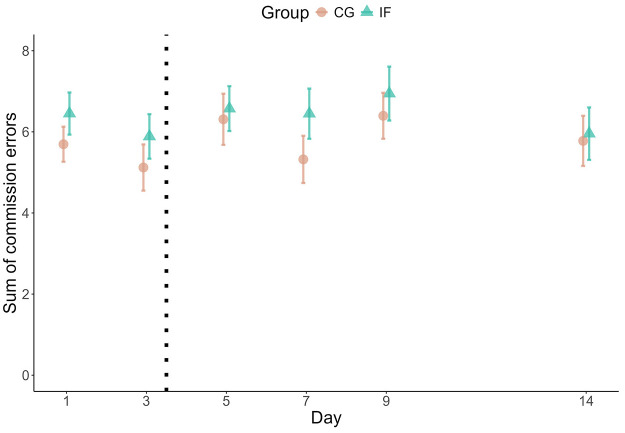
Median number of errors per measurement day. The plot shows the cumulated errors as points and their Standard Errors as error bars.

We modelled participants’ number of errors as an aggregated binomial model (Table 3; Supplemental Figure S2). The fasting group did not make substantially more or fewer errors overall, as indicated by the large Bayesfactor in favour of the model without group differences, *BF_01_* = 10,862 (note that the subscript 01 in *BF_01_* indicates evidence for M0 over M1; error rate fasting group: *M* = 6.37, *SD* = 4.50; control group: *M* = 5.79, *SD* = 4.38). This did not change over the course of the intervention (Figure 1). Notwithstanding, there was substantial heterogeneity between participants, as indicated by the variance between participants’ intercepts (*SD* = 0.74; *IQR* = [0.27, 0.63], 95% CI = [0.12, 1.30]; Supplemental Figure S3) and the estimated individual trajectories (*SD* = 0.30; *IQR* = [0.87, 1.12], 95% CI = [0.63, 1.45]). More flexible models – linear basis splines and cubic regression – also estimated a flat trajectory of performance over time and no group differences (Supplemental Figures S4 and S5). Furthermore, post-error slowing did not differ between fasting and control group (*B IF* < −0.01, *SE* = 0.01, 95% CI = [−0.02, 0.01]). In sum, cognitive performance was similar in both groups.

**Table 3.** Coefficients of objective cognitive performance model.

Variable	Coefficient (odds-scale)	SE	95% CI
Intercept	0.40	1.12	[0.32, 0.50]
IF	0.99	1.18	[0.71, 1.36]
Baseline covariate	1.23	1.02	[1.19, 1.29]
Repetition	0.99	1.06	[0.89, 1.09]
IF: repetition	1.00	1.08	[0.86, 1.16]

### Moderators of objective cognitive performance

We tested whether moderators could explain the observed differences between participants (see Supplemental Material for the complete modelling results). Higher *subjective* cognitive performance was associated with slightly lower error rates (*B subj cog perf* = 0.99, *SE* = 1.00, 95% CI = [0.99, 1.00]), however, without group differences (*B subj cog perf : group* = 1.00, *SE* = 1.00, 95% CI = [0.99, 1.01]). We did not observe effects of the moderators positive or negative mood, age, sex, BMI, prior expectations of the effects of fasting (Supplemental Tables S2–S6) or trait questionnaires.

#### Did objective cognitive performance differ between groups?

Error rates on the between-groups tasks were modelled as Poisson distributions. There were no substantial differences between groups in Simon task error rates on day 5 (*BF_01_* = 45.95; Supplemental Figure S6), Flanker task error rates on day 7 (*BF_01_* = 27.13) or Stroop task error rates on day 9 (*BF_01_* = 54.79).

### Did fasting influence participants’ subjective cognitive performance?

**Figure 2.**
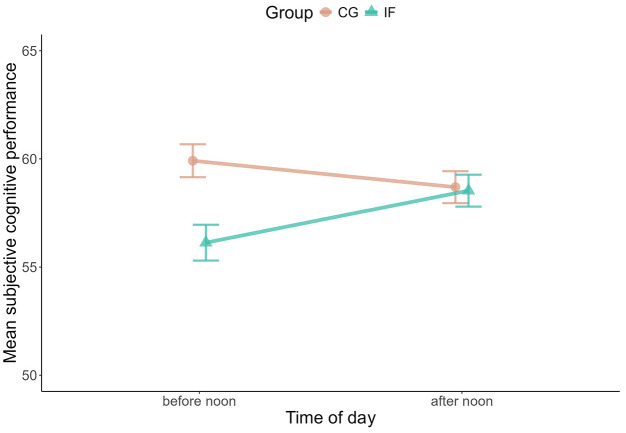
Changes in mean subjective cognitive performance within days. The plot shows the difference between mean subjective cognitive performance before and after noon for both groups.

Next to objectively measured cognitive performance, we also asked participants to indicate subjectively how well they performed before (fasted) and after noon (having broken the fast) on each study day. We did not observe changes between groups over the days of the study (Supplemental Tables S7 and S8) but when comparing before noon to after noon: The self-reported cognitive performance increased in the fasting group from before to after noon whereas it slightly decreased in the same time-window for the control group (Bayes Factor for the interaction model IF × afternoon versus the additive model IF + afternoon: *BF_10_* = 44.36; *B IF* = −3.12, *SE* = 1.90, 95% CI = [−6.83, 0.65]; *B IF: afternoon* = 3.22, *SE* = 1.25, 95% CI = [0.72, 5.68]; Figure 2). There was substantial variance between participants’ subjective cognitive performance before noon (varying intercepts *SD* = 10.16; *SE* = 0.77, 95% CI = [0.67, 0.02]) and to a smaller degree between the change to the afternoon assessment (varying slope *SD* = 0.63, *SE* = 0.67, 95% CI = 0.02, 2.61]).

#### Moderators of subjective cognitive performance

Positive mood explained some of the differences between groups in their subjective cognitive performance: the association between positive mood and subjective cognitive performance (B positive mood = 0.49, SE = 0.04, 95% CI = [0.42, 0.56]) was 0.13 units higher in the fasted group compared to control group on the 0–100 VAS (SE = 0.05, 95% CI = [0.04, 0.22]). Negative mood did not show any association (Supplemental Table S9). Expectations were also not associated with subjective cognitive performance in the fasted group (Supplemental Table S12).

### Subjective mood

**Figure 3.**
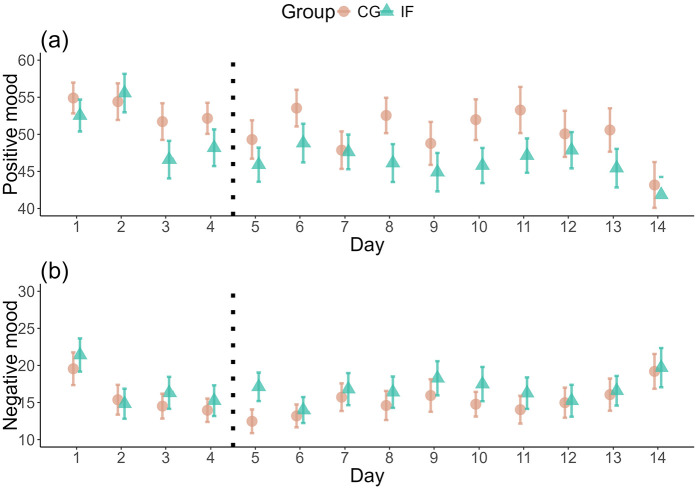
Difference in positive and negative mood over time. The plot shows the self-reported positive (a) and negative (b) mood before noon, that is, when the IF group was fasting, over the course of the study. The y-axis starts at 40 (a) and 10 (b) for better visibility. The dotted vertical line denotes the start of the intervention. Mean values and their Standard Errors are shown.

Considering mood as an outcome, there were no substantial differences between fasting and control group in positive mood (BF_01_ = 1.15 × e^113^; IF: M = 45.86, SD = 18.52; CG: M = 48.90, SD = 20.64) or negative mood (BF_01_ = 2985.91; IF: M = 17.13, SD = 16.88; CG: M = 15.46, SD = 14.95) during the daily fasting period (Supplemental Table S13, Figure 3).

## Discussion

In this randomised controlled trial, we investigated whether intermittent fasting negatively impacts cognition and mood using a Bayesian modelling approach. Participants completed daily questionnaires and cognitive tests on their personal devices, allowing us to assess effects in real-world settings. Overall, we found evidence that fasting does *not* impair objectively measured cognitive performance or mood within the first 10 days of fasting. However, participants consistently reported feeling less concentrated while fasting compared to after breaking the fast. Positive mood was more strongly positively associated with subjective cognitive performance in the fasting, compared to control group. Other moderators failed to explain the variance between individuals. These findings have two key implications: First, the relationship between energy intake and cognitive function should not be reduced to metabolic mechanisms only, suggesting a more prominent role of psychological factors. Second, intermittent fasting appears to be a viable lifestyle or health intervention, even for those engaged in cognitively demanding tasks. We explore these implications below.

### Through which mechanism could IF affect cognitive performance?

Our study addressed concerns raised by previous research that links breakfast skipping to cognitive impairments (Benau et al., 2021; Galioto and Spitznagel, 2016). We consider three possible mechanisms of this link here – energy availability, mood changes and expectation effects – which will be discussed in turn. The brain has a high energy demand (Göbel et al., 2013; Mergenthaler et al., 2013) and limited energy storage capacity (Brocchi et al., 2022). Restricting energy supply through fasting could therefore limit cognitive function. This has been observed in some studies using single overnight fasting interventions (Owen et al., 2012; Watkins and Serpell, 2016). Yet, the brain has privileged access to energy resources (Mergenthaler et al., 2013) and ketones serve as substitute energy source when glucose levels run low (Longo et al., 2021; Mattson et al., 2017; Wilhelmi de Toledo et al., 2020). Additionally, energy depletion appears to have broad, non-specific effects on cognition rather than targeting specific cognitive domains (Ampel et al., 2018). Taken together, brief (i.e. up to 18 hours)-fasting does not necessarily have detrimental effects on cognitive function due to a lack of available energy.

Considering long-term effects of IF, animal studies indicate that IF increases brain-derived neurotrophic factor expression and shields neurons from excitotoxic damage, thereby promoting synaptic plasticity and resilience (Anson et al., 2003; Rothman and Mattson, 2013). Furthermore, ketones as fuel for the brain may lower oxidative stress and support mitochondrial function (Mattson et al., 2017). Together, these neuronal mechanisms imply positive effects on cognition. These neurobiological changes may, in principle, translate into cognitive gains – yet they unfold over weeks, well beyond our 10-day IF intervention window. In the short term, quicker-acting psychological factors, notably mood and expectations, are therefore more likely to shape performance, a possibility we explored next to explaining? our null effects.

Mood changes have also been proposed as a mechanism linking breakfast skipping to cognitive performance (Adolphus et al., 2016). In our study, positive mood moderated subjective cognitive performance overall and this association was higher in fasted participants. Yet, we observed no meaningful differences in positive or negative mood between intervention groups over time. Furthermore, existing evidence on the mood effects of IF remains mixed. For instance, females on an Alternate Day Fasting (ADF) regimen reported increased distraction and negative mood (Appleton and Baker, 2015), whereas other studies found reductions in depression and anxiety (Kessler et al., 2018) or decreases in anger and aggression in older males (Hussin et al., 2013). Fasting may affect subjective cognitive performance through changes in mood. However, further research that, for example, manipulates mood and tests effects on subjective cognitive performance, is necessary to establish a causal link.

Study participants’ expectations may also play a role: individuals may expect fasting to impair cognitive performance, with subsequent effects on their behaviour. Experimental evidence supports this, showing that expectations regarding effects of breakfast-skipping on cognitive performance can be induced, with measurable changes in objectively measured cognitive performance (Bamberg and Roefs, 2021). Placebo interventions have been used to manipulate cognitive performance in other contexts with mixed success (Hoffmann et al., 2018; Parong et al., 2022; Sinke et al., 2016; Winkler and Hermann, 2019). Thus, expectations, when manipulated experimentally, may influence cognitive performance. Yet, in our study, participants’ observed expectations regarding the fasting intervention’s effects did not show any relation with cognitive performance. Thus, naturally occurring expectations – as opposed to manipulated – regarding an intervention may not be strong enough to substantially impact the outcome. In summary, the pathways through which fasting may affect cognitive performance remain unclear, necessitating further research with a focus on testing these different mechanisms.

#### What are the implications for IF as a health behaviour?

Considering the popularity of IF for its health benefits and for weight-loss, it is important to explore potential side-effects and challenges to adherence, such as detriments to cognitive performance or mood, in a naturalistic setting. Fasted participants performed objectively as well as control participants, indicating that IF can be taken up without worry about cognitive detriments. The stable mood observed in our study may support adherence to intermittent fasting. Negative mood effects could undermine a practitioner’s ability and motivation to stick to the protocol. For instance, reduced adherence by participants experiencing lower mood has been reported in exercise interventions for weight-loss (Burgess et al., 2017) or recovery after musculoskeletal injury (Hui et al., 2023). Since mood remained stable in our study, it does not pose a challenge to IF adherence. Notwithstanding, despite promising findings regarding adherence based on mood, the lowered subjective cognitive performance while fasting might affect maintenance or acceptance of the regimen. Healthcare professionals may consider this absence of mood-related side effects when recommending IF to potential users.

### Limitations

There are several points of the study design to be considered as potential limitations. We timed the objective cognitive performance measures to be at the end of the fasting window, expecting strongest effects there and focusing more on potential changes from day to day than within day. Given the subjective cognitive performance change from before to after breaking the fast, future studies may want to focus more on within-day-changes in objective cognitive performance. Although adding more task repetitions could have provided additional data, we aimed to minimise participant burden to maintain engagement, also reducing unwanted training effects. With online cognitive tasks, there is always some amount of unexplained noise in the data. However, the response patterns, with generally high performance, response times in the to-be-expected range and few missing responses, indicate that participants overall attended to the task. Moreover, the uncontrolled testing environment, while noisier, mirrors real-world conditions better.

For subjective reports, questions arise about participants’ ability to perceive internal signals like hunger, fatigue, mood, or concentration. Hunger peaked as expected during fasting, but other associations were unclear, highlighting the need for refined measures in ambulatory studies on interoception.

Furthermore, despite the indications that participants followed the fasting intervention, such as reasonable reported durations, associations with hunger and qualitative feedback regarding their experience with the intervention, we cannot say with complete certainty, that participants followed the intervention protocol completely. Similarly, we cannot be certain that the control group ate their usual meals and did not fast, contrary to their instructions. Notwithstanding, the substantially lower hunger levels of the control group indicates that they followed the instructions. Furthermore, the control group received advice for fasting with their debriefing and was informed of this at the beginning of the study, to further increase their compliance with the study protocol. Objective measurements like continuous blood-glucose monitoring or repeated breath-ketone measurements could have theoretically helped to monitor intervention adherence. Yet, this would have incurred additional participant burden.

To further advance research on IF’s effects on cognition, future studies may prefer to focus on higher resolution within days and less over the course of days. Moreover, other fasting protocols like ADF, which may also be quite demanding for the individual since it requires fasting one complete day may be worth investigating. Furthermore, investigating other cognitive domains, such as working memory, or trying to find measures that are more strongly related to actual (work-) performance may also increase the breadth and relevance of this research. Based on our findings, there are no reservations regarding IF as a healthy eating behaviour from a cognitive and psychological standpoint.

## Supplemental Material

sj-pdf-1-hpq-10.1177_13591053251351204 – Supplemental material for Stable cognitive performance while adapting to intermittent fasting: A randomised controlled trial Supplemental material, sj-pdf-1-hpq-10.1177_13591053251351204 for Stable cognitive performance while adapting to intermittent fasting: A randomised controlled trial by Christoph Bamberg, Julia Reichenberger and Jens Blechert in Journal of Health Psychology
