# Supplementary material for: Stable cognitive performance while adapting to intermittent fasting: A randomised controlled trial
Source: J Health Psychol. 2025 Jul 23;31(4):1362–77. doi: 10.1177/13591053251351204 (PMC12960773; doi:10.1177/13591053251351204)
Supplement: sj-pdf-1-hpq-10.1177_13591053251351204 – Supplemental material for Stable cognitive performance while adapting to intermittent fasting: A randomised controlled trial [file sj-pdf-1-hpq-10.1177_13591053251351204.pdf]

Supplemental Material Stable cognitive performance while adapting to intermittent fasting

Supplemental Methods

List of used trait questionnaires

Composite Scale of Morningness reduced, CSM-r (Randler, 2014), Salzburg Stress Eating Scale, SSES (Meule et al., 2018b), Salzburg Emotional Eating Scale, SEES (Meule et al., 2018a), Dutch Eating Behaviour Questionnaire, DEBQ (Van Strien et al., 1986), Eating Disorder Examination Questionnaire, EDE-Q (Hilbert & Tuschen-Caffier, 2016), Intuitive Eating Scale 2, IES-2 (Tylka & Kroon Van Diest, 2013), Mindful Eating Behaviour Scale, MEBS (Winkens et al., 2018), International Physical Activity Questionnaire, IPAQ (Craig et al., 2003),Willpower Scale (Job et al., 2010), Pittsburgh Sleep Quality Index, PSQI (Buysse et al., 1989). After the last day of the intervention, participants answered the PSQI again for the 14-day study period, cycle-related questions for women and their subjective experience in the study.

Priors in the Bayesian models

For the aggregated binomial model of the within-individual changes of commission errors, we used the following priors: Intercept: Normal(location or mean = 0, scale or SD = 10), coefficients: Normal(location = 0, scale = 10), SD for grouped effects: Half-Cauchy(scale = 3), correlation between grouped effects: Lewandowski-Kurowicka-Joe (LKJ) Cholesky(scale = 1).

For the Poisson models of between-groups error rates, we used these priors: Intercept: Normal(location or mean = 10, scale or SD = 10), coefficients: Normal(location = 0, scale = 10), SD for grouped effects: Half-Cauchy(scale = 3), correlation between grouped effects: Lewandowski-Kurowicka-Joe (LKJ) Cholesky(scale = 1).

For models with smartphone questionnaire-based data, i.e., subjective cognitive performance and mood, modelled with Normal regression models, we used these priors: Intercept: Normal(location = [at the mean of the data], scale = 10), Sigma: Half-Cauchy(scale = 3), SD for grouped effects: Half-Cauchy(scale = 3), correlation between grouped effects: Lewandowski-Kurowicka-Joe (LKJ) Cholesky(scale = 1).

Finally, for the pooled correlation in the multi-level correlation models, we used Normal(location= 0, scale = 1) priors. See below for a prior sensitivity analysis.

Supplemental Results

Figure S1.

Participant retention flow chart

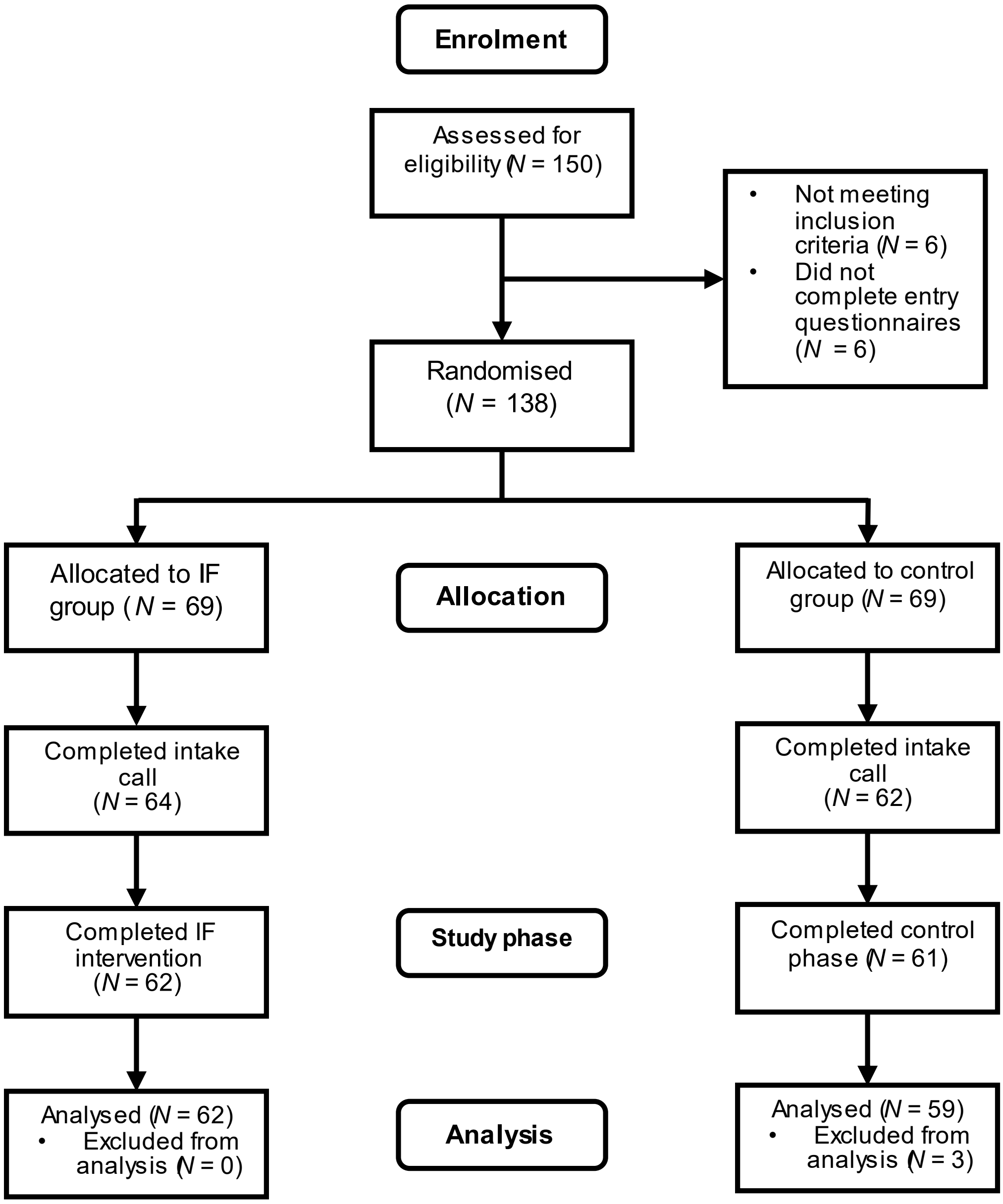

Task sensitivity checks for between-groups tasks

For the between-person tasks, we confirmed that responses were faster when stimuli were presented with congruent flankers in the Flanker task (BF_{10} = 1.01*e^8; M congruent RT = 0.45, SD = 0.08; M incongruent RT = 0.51, SD = 0.10) or congruent colour in the Stroop task (BF_{10} = 54.61; M congruent RT = 0.60, SD = 0.14; M incongruent RT = 0.64, SD = 0.15). We could not confirm this for the Simon task (BF_{10} = 1.98; M congruent RT = 0.44, SD = 0.11; M incongruent RT = 0.46, SD = 0.10)

Further results for within-individuals task

Figure S2.

Posterior densities and trace plots for the main commission error model

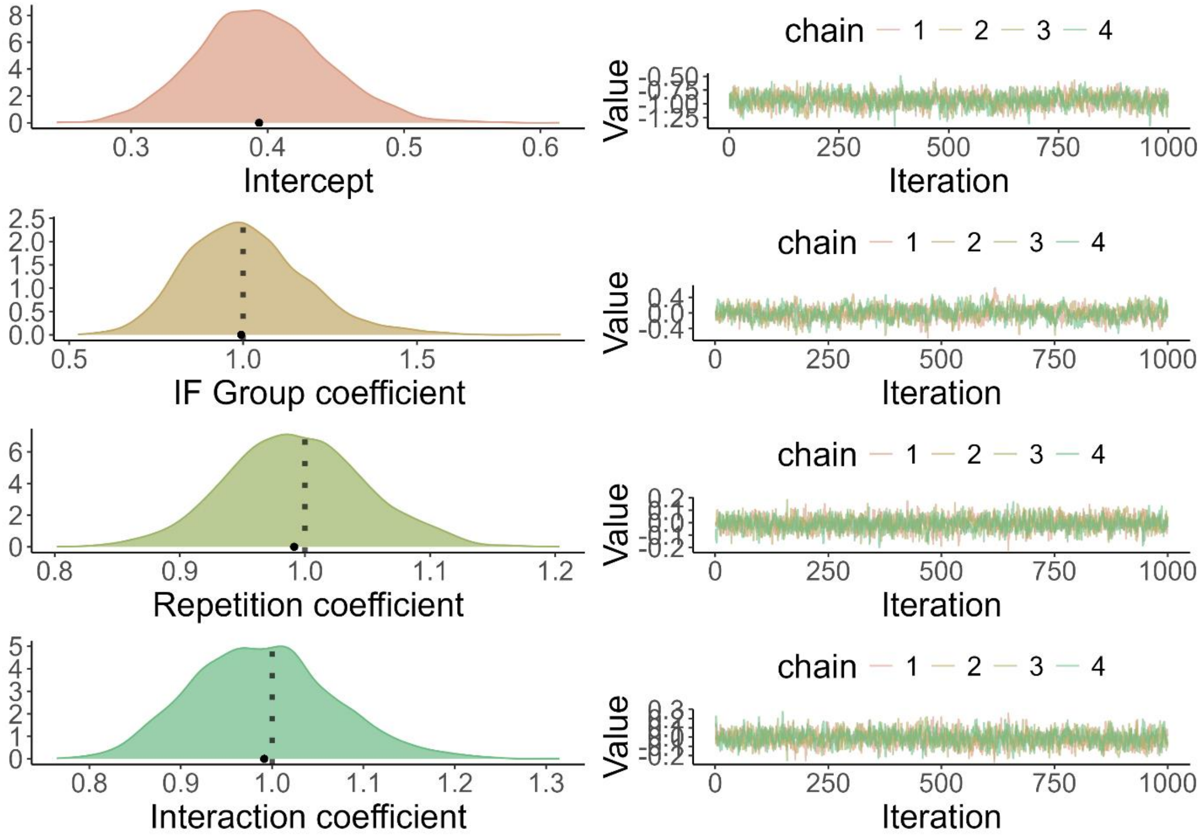

Figure S3.

Individual Differences in Performance

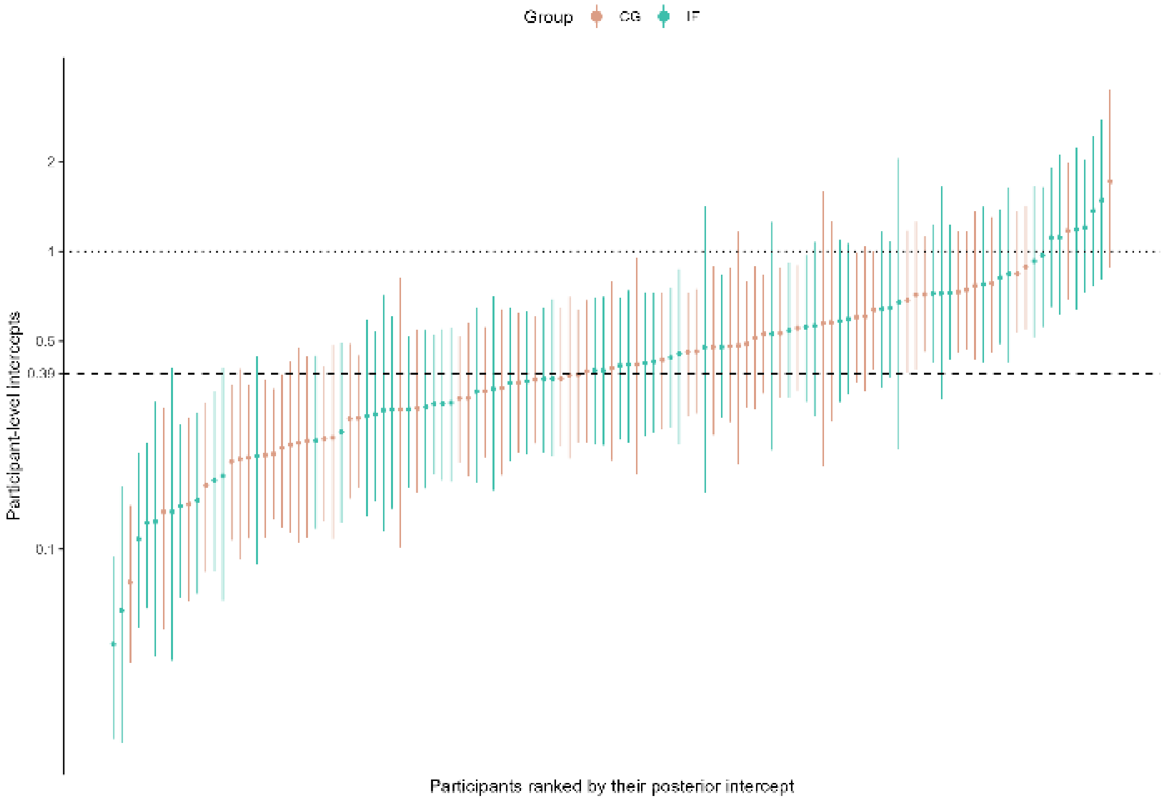

Note: The plot shows the intercepts, as the estimated posterior odds of making an error, for each participant. The dashed line shows the median posterior error rate. The dotted line shows equal odds of committing and not committing an error.

Basis-spline regression for objective cognitive performance

We used basis spline regression to account for different intervals between the first five and last repetition. The model includes three regression functions: baseline period (repetitions 1-2), first half of the intervention (repetitions 2-5), and last half (repetitions 5-6). Figure S4 shows the estimated regression spline for the intervention group. Despite higher degrees of

freedom, the trajectory remains relatively flat and does not improve data explanation over a simple linear model (elpdf difference[1]: linear – spline = -1.8, SE difference = 6.4).

Figure S4.

Basis Spline Regression for Intervention Group

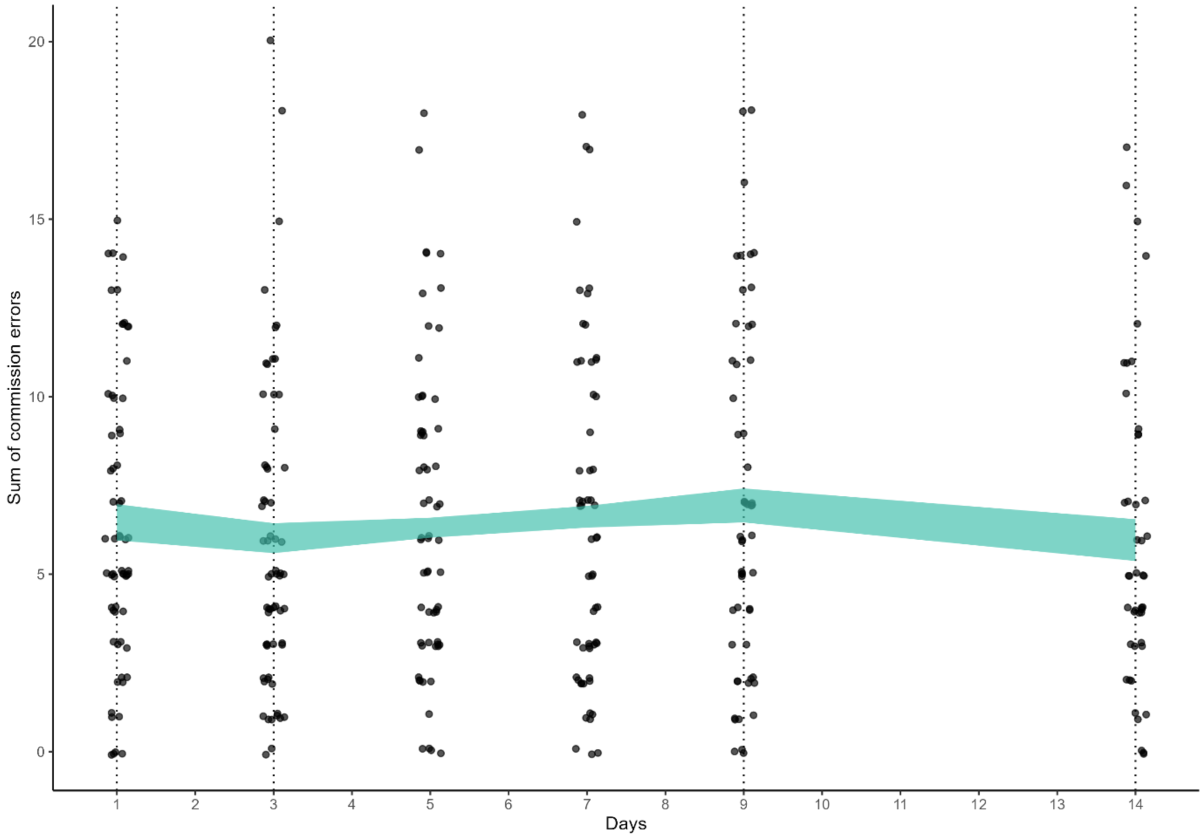

Note: The x-axis shows the days of the study to better represent the gap between measurements. The vertical dotted lines indicate the knots of the basis spline model. The y-axis shows the number of commission errors made per assessment. The ribbon shows the 95% highest density interval. The dashed line shows a simple linear model for comparison.

[1] We used leave-one-out cross-validation with pareto-smoothed importance sampling to compare the fit of different models. The fit characteristics of each model are expressed as the expected log pointwise predictive density (elpdf). We calculate the difference between elpdfs for both models. If this difference is orders of magnitude larger than its Standard Error, it can be interpreted as a meaningful difference in fit.

Polynomial Model

Moreover, a third order polynomial regression model did not explain the error rates better than the linear model (elpdf difference linear model – polynomial model = - 2.5, SE of the difference = 5.3).

Figure S5.

Conditional effects plot polynomial regression for objective cognitive performance over time

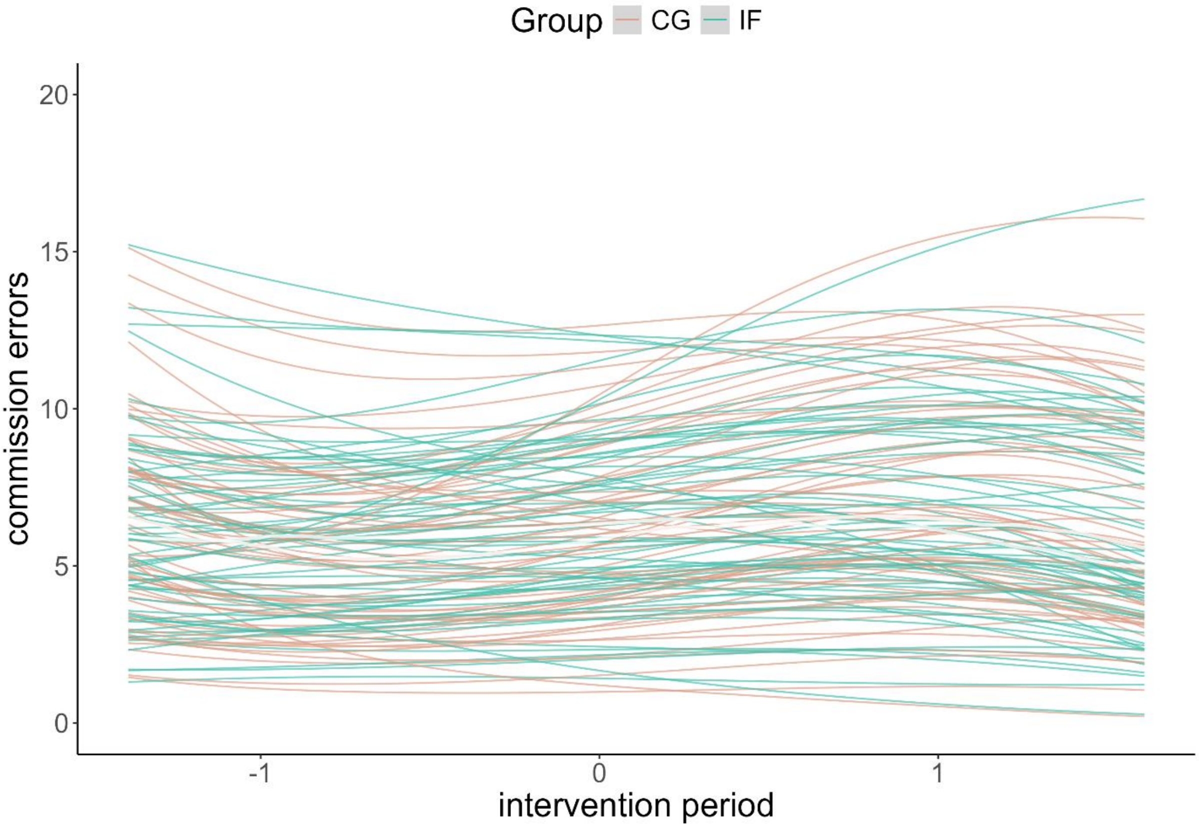

Note: the measurement repetition term was standardised to improve the model fit.

Moderators of within-individual objective cognitive performance

Table S1.

Subjective cognitive performance moderator for cognitive performance model

	Coefficient (odds-scale)	SE	95% CI
Intercept	0.65	1.21	[0.45, 0.94]
IF	0.96	1.30	[0.57, 1.61]
Baseline covariate (centred)	1.24	1.02	[1.20, 1.30]
Subjective cog. perf.	0.99	1.00	[0.99, 1.00]
IF : subjective cog. perf.	1.00	1.00	[0.99, 1.01]

Table S2.

Negative mood moderator for cognitive performance model

	Coefficient (odds-scale)	SE	95% CI
Intercept	0.41	1.13	[0.32, 0.53]
IF	0.89	1.19	[0.63, 1.25]
Baseline covariate (centred)	1.25	1.02	[1.20, 1.30]
Negative mood	1.00	1.00	[0.99, 1.05]
IF : negative mood	1.01	1.01	[0.99, 1.02]

Table S3.

Negative mood moderator for cognitive performance mode

	Coefficient (odds-scale)	SE	95% CI
Intercept	0.50	1.21	[0.35, 0.73]
IF	1.03	1.30	[0.61, 1.74]
Baseline covariate (centred)	1.25	1.02	[1.20, 1.30]
Positive mood	0.99	1.00	[1.00, 1.00]
IF : positive mood	1.00	1.00	[0.99, 1.01]

Table S4.

Moderator age for cognitive performance model

	Coefficient (odds-scale)	SE	95% CI
Intercept	0.58	1.37	[0.32, 1.09]
IF	0.53	1.60	[0.20, 1.30]
Baseline covariate (centred)	1.25	1.02	[1.25, 1.32]
Age	0.98	1.01	[0.96, 1.00]
IF : age	1.02	1.02	[0.99, 1.06]

Table S5.

Moderator BMI for cognitive performance model

	Coefficient (odds-scale)	SE	95% CI
Intercept	0.48	1.99	[0.13, 1.99]
IF	0.72	2.72	[0.10, 5.46]
Baseline covariate (centred)	1.29	1.02	[1.26, 1.33]
BMI	0.99	1.03	[0.93, 1.05]
IF : BMI	1.01	1.04	[0.93, 1.10]

Table S6.

Moderator Hunger expectations for cognitive performance model

	Coefficient (odds-scale)	SE	95% CI
Intercept	0.36	1.21	[0.25, 0.52]
IF	0.83	1.30	[0.50, 1.38]
Baseline covariate (centred)	1.30	1.02	[1.26, 1.33]
Hunger exp.	1.00	1.00	[0.99, 1.01]
IF : hunger exp.	1.00	1.00	[0.99, 1.01]

Further results between participants tasks

The coefficients for group assignment in the models are Simon: B IF = 1.19, SE =1.16, 95%CI = [0.89, 1.57]; Flanker: B IF = 1.31, SE = 1.24, 95%CI = [0.85, 2.04]; Stroop: B IF = 0.97, SE = 1.17, 95%CI = [0.72, 1.34].

Figure S6.

Error rates in between-groups tasks

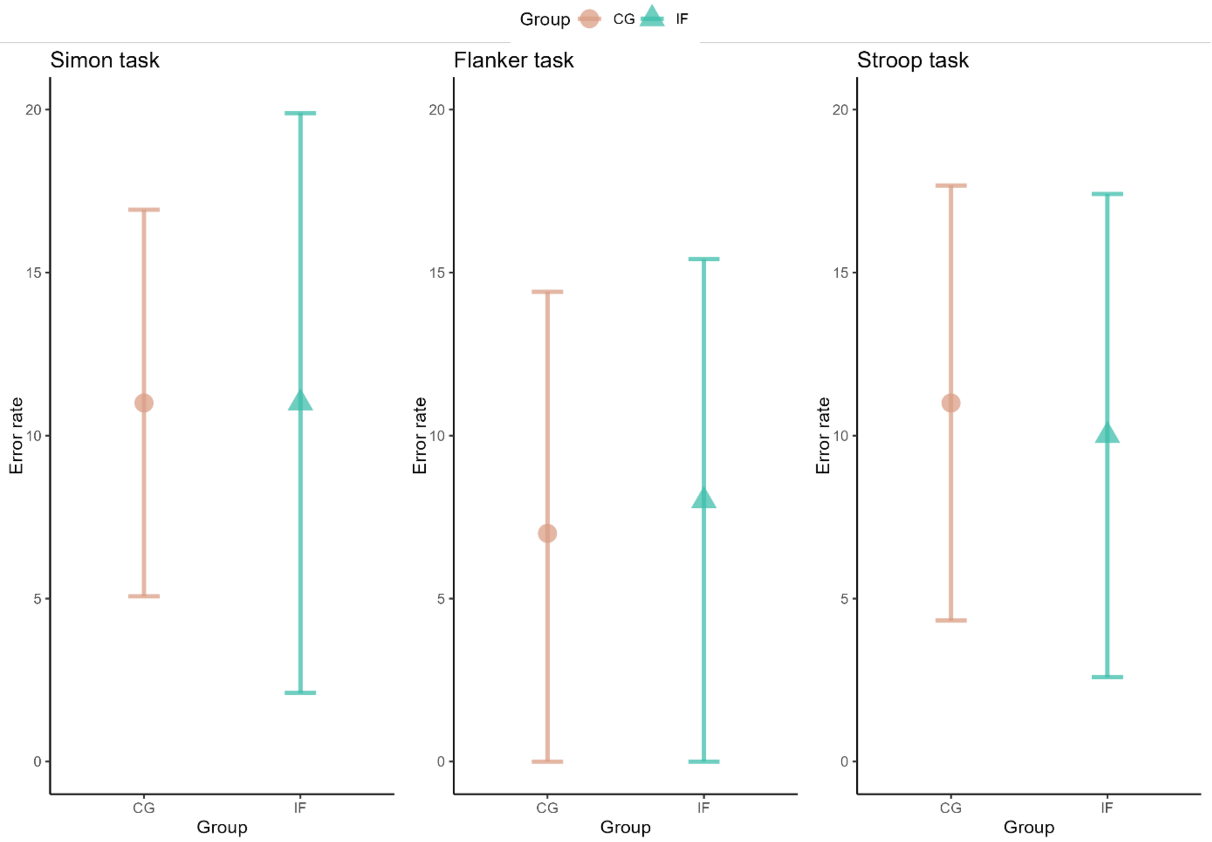

Note: The plot shows the median error rates for control- and fasting participants for the three tasks. Error bars are based on the MAD.

Further results subjective cognitive performance

This is a complementary perspective to our main analysis of subjective cognitive performance. When considering the fasted, before noon measurement, fasting participants

reported insignificantly lower cognitive performance compared to control (BF_{01} = 1.98; Table S7; Figure S7A). This difference did not change over time. Fasting and control group did not differ in the afternoon measurements (Table S8; Figure S7B). The sleepiness before or after noon, as a complementary measure to concentration, did not differ between groups as well, without change throughout the assessment period either (Table S9).

Table S7.

Coefficients of Subjective Cognitive Performance Before Noon

	Coefficient	SE	95% CI
Intercept	59.72	2.23	[55.26, 64.09]
IF	-5.68	2.82	[-11.16, -0.16]
Baseline covariate (centred)	0.59	0.07	[0.45, 0.72]
Day	-0.07	0.25	[-0.56, 0.42]
IF : Day	0.35	0.32	[-0.28, 0.99]

Table S8.

Coefficients of Subjective Cognitive Performance Model in Afternoon

	Coefficient	SE	95% CI
Intercept	56.33	2.16	[52.10, 60.63]
IF	0.71	2.74	[-4.67, 6.03]
Baseline covariate (centred)	0.59	0.07	[0.45, 0.72]
Day	0.19	0.23	[-0.25, 0.64]
IF : Day	0.01	0.29	[-0.56, 0.58]

Figure S7.

Change in subjective performance over time

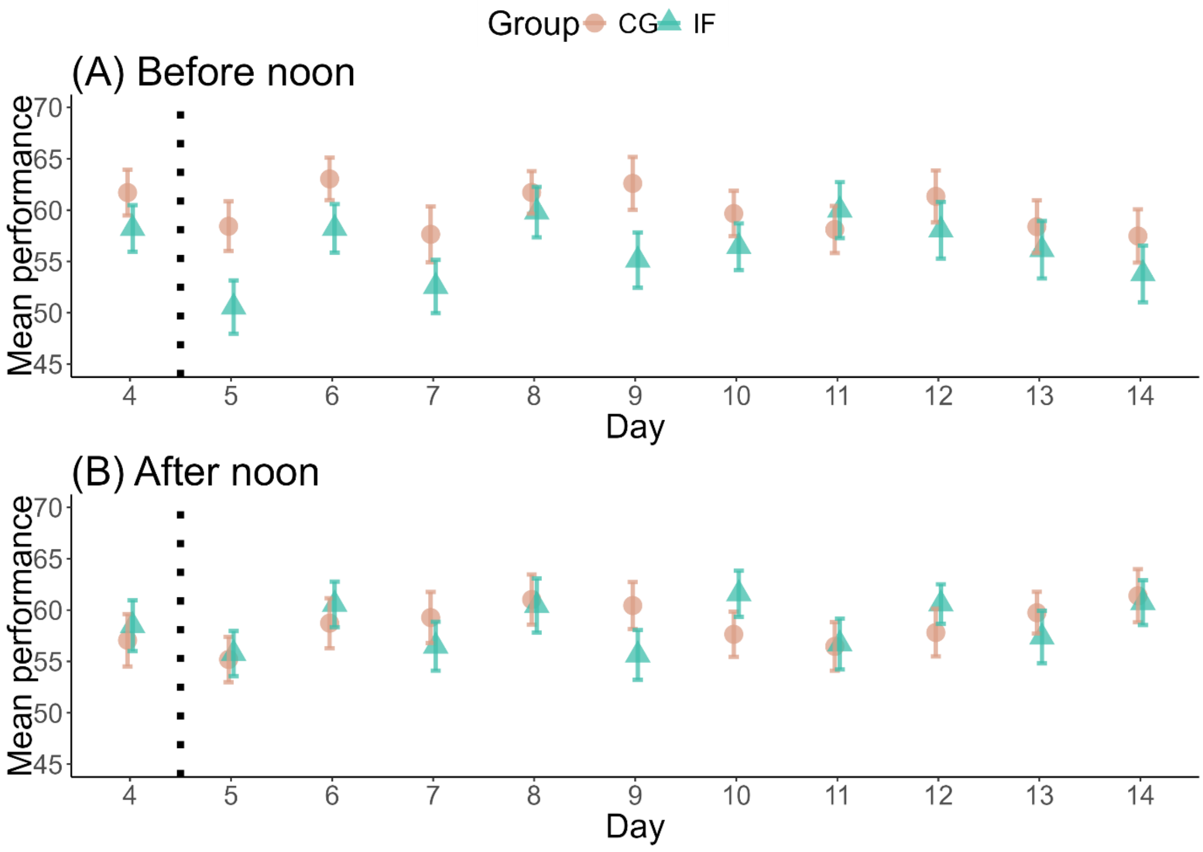

Note: The y-axis starts at 50 for better visibility. The dotted vertical line denotes the start of the intervention. Mean values and their Standard Errors are shown.

Table S8.

Coefficients of daytime sleepiness model

	Coefficient	SE	95% CI
Intercept	36.69	3.01	[30.71, 42.60]
IF	0.59	3.49	[-6.20, 7.54]
Baseline covariate (centred)	0.54	0.08	[0.38, 0.69]
Day	-0.01	0.30	[-0.60, 0.57]
IF : day	0.28	0.37	[-0.44, 0.98]

Results moderators subjective cognitive performance

Table S9

Model Results for Negative and Positive Mood Model

	Coefficient	SE	95% CI
	Positive mood factor		
Intercept	34.67	1.96	[30.83, 38.51]
Baseline covariate (centred)	0.21	0.06	[0.08, 0.33]
IF	-7.55	2.48	[-12.45, -2.64]
Pos. mood	0.49	0.04	[0.42, 0.56]
IF : pos. mood	0.13	0.05	[0.04, 0.22]
	Negative mood factor		
Intercept	63.32	1.36	[60.63, 66.00]
Baseline covariate (centred)	0.45	0.07	[0.32, 0.59]
IF	-1.17	1.88	[-4.85, 2.52]
Neg. mood	-0.26	0.06	[-0.37,-0.15]
IF : neg. mood	-0.12	0.08	[-0.27,0.03]

Table S10.

Model results for gender differences and subjective cognitive performance

	Coefficient	SE	95% CI
Intercept	57.41	2.30	[52.90, 62.04]
IF	-0.62	2.63	[-5.76, 4.70]
Baseline covariate (centred)	0.59	0.07	[0.45, 0.73]
Gender female	2.42	2.40	[-2.34, 7.13]
IF : gender female	-3.37	2.81	[-9.09, 2.08]

Table S11.

Model results for age moderator and subjective cognitive performance

	Coefficient	SE	95% CI
Intercept	55.55	4.00	[47.68, 63.22]
IF	-1.82	4.21	[-9.77, 6.51]
Baseline covariate (centred)	0.58	0.07	[0.45, 0.71]
Age	0.15	0.15	[-0.14, 0.44]
IF : age	-0.06	0.16	[-0.38, 0.25]

Table S12.

Model results for hunger expectations moderator and subjective cognitive performance, for fasted participants

	Coefficient	SE	95% CI
Intercept	54.74	2.95	[49.03, 60.71]
Baseline covariate (centred)	0.65	0.10	[0.46, 0.84]
Expectations	0.03	0.05	[-0.08, 0.12]

Supplemental results for change in mood over time

Table S13.

Model Results for Negative and Positive Mood Model

	Coefficient (metric scale)	SE	95% CI
	Positive mood		
Intercept	50.44	2.06	[46.43, 54.57]
IF	-1.56	2.81	[-7.11, 3.89]
Baseline covariate (centred)	0.96	0.07	[0.82, 1.10]
Day	-0.02	0.25	[-0.49, 0.47]
IF : Day	-0.21	0.33	[-0.85, 0.44]
	Negative mood		
Intercept	12.31	1.58	[9.25, 15.44]
IF	2.51	2.16	[-1.73, 6.78]
Baseline covariate (centred)	0.77	0.05	[0.68, 0.87]
Day	0.31	0.18	[-0.04, 0.65]
IF : Day	-0.13	0.25	[-0.62, 0.35]

Sensitivity analyses

Figure S8.

Posterior density of interaction in objective cognitive performance model

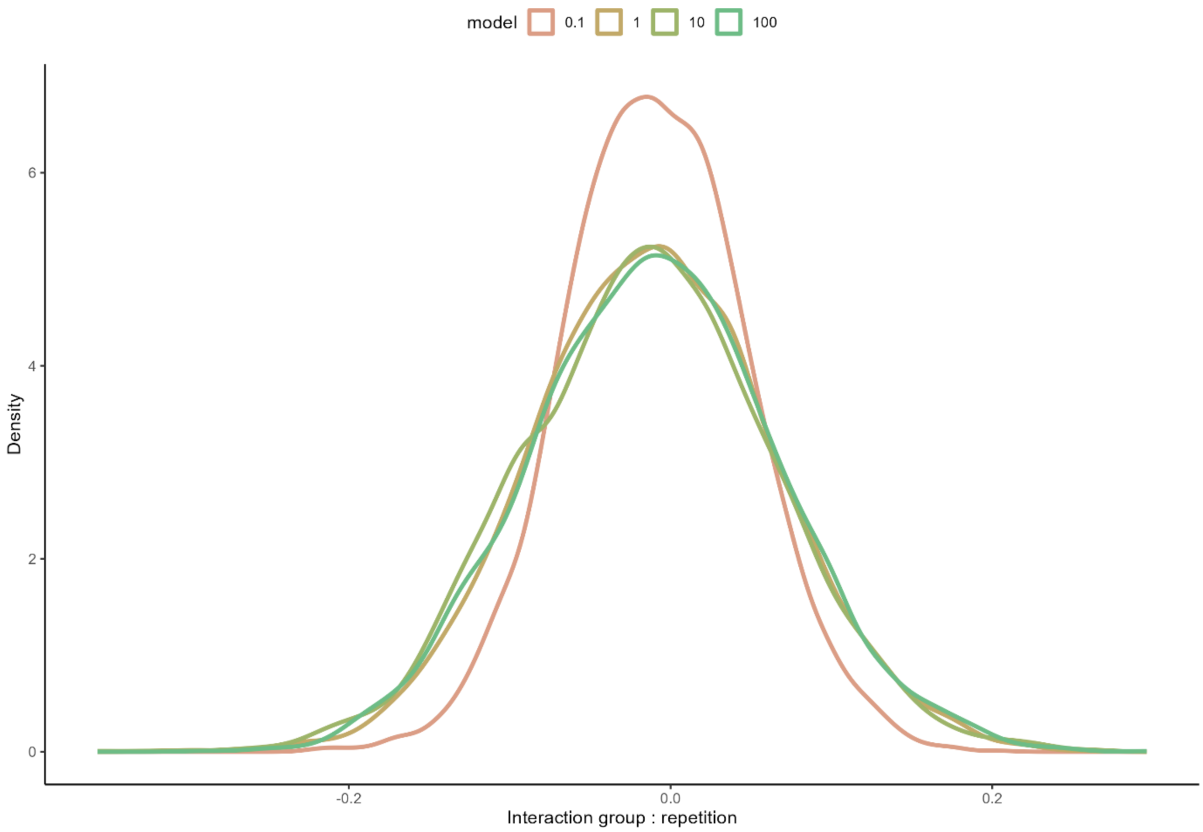

Note: the standard deviation for the prior of all coefficients was varied between 0.1, 1, 10 and 100. Thus, the prior confidence in the interaction between group assignment and measurement repetition varied.

Figure S9.

Posterior density of interaction in subjective cognitive performance model

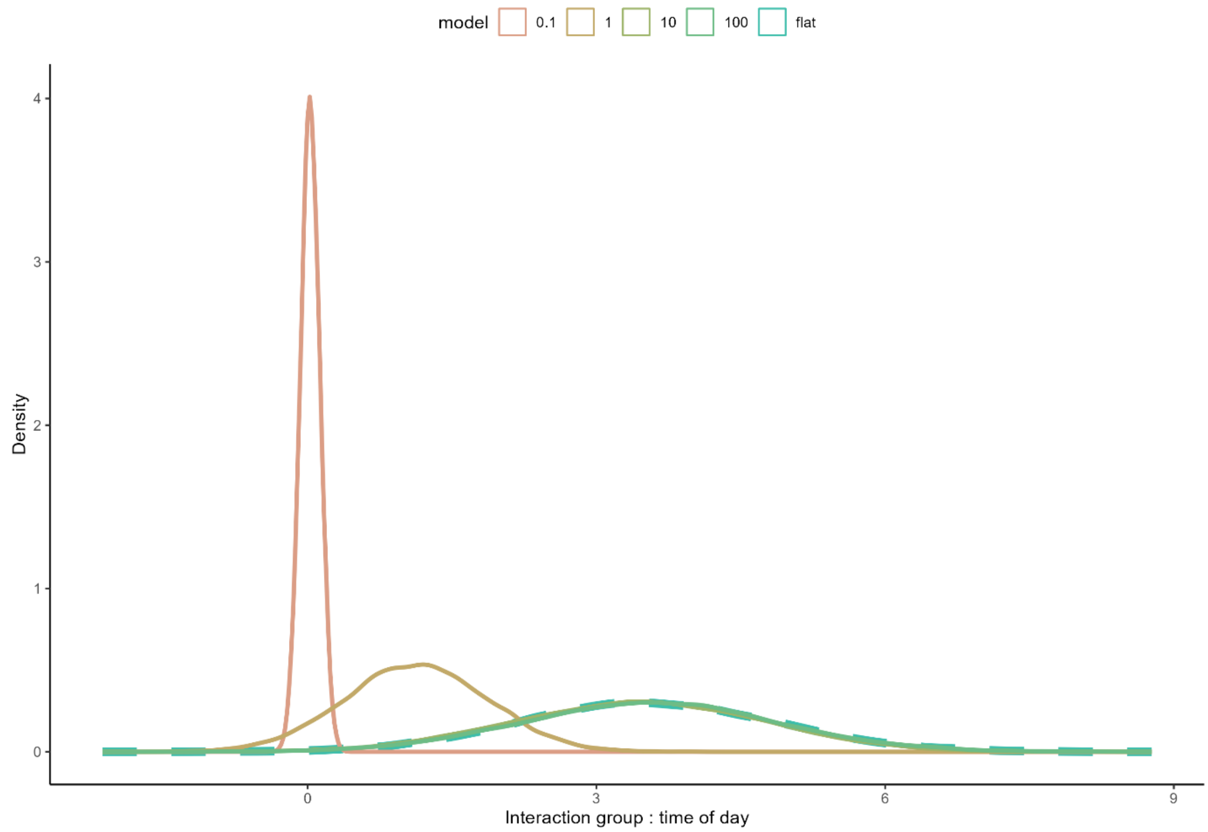

Note: the standard deviation for the prior of all coefficients was varied between 0.1, 1, 10 and 100. Thus, the prior confidence in the interaction between group assignment and measurement repetition varied. In addition, the default flat prior was added as a dashed blue line

Unlike for the objective cognitive performance model, the priors of the subjective cognitive performance model seem to yield a substantial influence on the posterior estimates. In the main text, we report results with the prior Normal (mean = 0, SD = 10), corresponding to the light-green line in Figure S9. The models with prior Normal (mean = 0, SD = 10) and Normal (mean = 0, SD = 100) and the flat prior yield the same posterior. This hints at the fact that if the prior is as unspecific as Normal (mean = 0, SD = 10) or more unspecific, the posterior is mostly determined by the likelihood of the data (confirmed by the fact that the posterior of the model with completely flat priors is visually identical). We believe that a

Normal (mean = 0, SD = 10) prior is an adequate a priori expectation – 65% of the data are expected to be within -20 to +20 – and that the more skeptical Normal (mean=0, SD = 1) prior – with 65% of the data expected within -2 to +2 – is too pessimistic. Thus, we report the model with Normal (mean=0, SD = 10) prior.

Supplemental References

Buysse, D. J., Reynolds, C. F., Monk, T. H., Berman, S. R., & Kupfer, D. J. (1989). The Pittsburgh sleep quality index: A new instrument for psychiatric practice and research. *Psychiatry Research*, *28*(2), 193–213. https://doi.org/10.1016/0165-1781(89)90047-4

Craig, C. L., Marshall, A. L., Sjöström, M., Bauman, A. E., Booth, M. L., Ainsworth, B. E., Pratt, M., Ekelund, U., Yngve, A., Sallis, J. F., & Oja, P. (2003). International Physical Activity Questionnaire: 12-Country Reliability and Validity: *Medicine & Science in Sports & Exercise*, *35*(8), 1381–1395. https://doi.org/10.1249/01.MSS.0000078924.61453.FB

Hilbert, A., & Tuschen-Caffier, B. (2016). *Eating Disorder Examination Questionnaire—Deutschsprachige Übersetzung, 2. Auflage* (2nd ed.). dgvt-Verlag. https://www.dgvt-verlag.de/e-books/2_Hilbert_Tuschen-Caffier_EDE-Q_2016.pdf

Job, V., Dweck, C. S., & Walton, G. M. (2010). Ego Depletion—Is It All in Your Head?: Implicit Theories About Willpower Affect Self-Regulation. *Psychological Science*, *21*(11), 1686–1693. https://doi.org/10.1177/0956797610384745

Meule, A., Reichenberger, J., & Blechert, J. (2018a). Development and Preliminary Validation of the Salzburg Emotional Eating Scale. *Frontiers in Psychology*, *9*, 88. https://doi.org/10.3389/fpsyg.2018.00088

Meule, A., Reichenberger, J., & Blechert, J. (2018b). Development and preliminary validation of the Salzburg Stress Eating Scale. *Appetite*, *120*, 442–448. https://doi.org/10.1016/j.appet.2017.10.003

Randler, C. (2014). *CSM - Composite Scale of Morningness—Deutsche Fassung*. https://doi.org/10.23668/PSYCHARCHIVES.6485

Tylka, T. L., & Kroon Van Diest, A. M. (2013). The Intuitive Eating Scale–2: Item refinement and psychometric evaluation with college women and men. *Journal of Counseling Psychology*, *60*(1), 137–153. https://doi.org/10.1037/a0030893

Van Strien, T., Frijters, J. E. R., Bergers, G. P. A., & Defares, P. B. (1986). The Dutch Eating Behavior Questionnaire (DEBQ) for assessment of restrained, emotional, and external eating behavior. *International Journal of Eating Disorders*, *5*(2), 295–315. https://doi.org/10.1002/1098-108X(198602)5:2<295::AID-EAT2260050209>3.0.CO;2-T

Winkens, L. H. H., Van Strien, T., Barrada, J. R., Brouwer, I. A., Penninx, B. W. J. H., & Visser, M. (2018). The Mindful Eating Behavior Scale: Development and Psychometric Properties in a Sample of Dutch Adults Aged 55 Years and Older. *Journal of the Academy of Nutrition and Dietetics*, *118*(7), 1277-1290.e4. https://doi.org/10.1016/j.jand.2018.01.015
